# A two-in-one expression construct for biophysical and structural studies of the human pregnane X receptor ligand-binding domain, a pharmaceutical and environmental target

**DOI:** 10.1107/S2053230X2500069X

**Published:** 2025-02-09

**Authors:** Coralie Carivenc, Guillaume Laconde, Pauline Blanc, Muriel Amblard, William Bourguet, Vanessa Delfosse

**Affiliations:** a Centre de Biologie Structurale (CBS), Univ. Montpellier, INSERM, CNRS, Montpellier, France; b IBMM, Univ. Montpellier, CNRS, ENSCM, Montpellier, France; Bristol-Myers Squibb, USA

**Keywords:** PXR ligand-binding domain, SRC-1 fusion, purification and crystallization

## Abstract

The ligand-binding domain (LBD) of the human nuclear receptor pregnane X receptor (PXR) is known to crystallize in two different crystal forms, *P*2_1_2_1_2_1_ or *P*4_3_2_1_2, depending on the construct and the strategy used for protein production, as well as the presence or absence of the coactivator-derived peptide SRC-1. In order to facilitate biophysical and structural studies, a versatile construct was designed that allows access to both forms. This was achieved by introducing a thrombin cleavage site between the PXR_LBD_ and the SRC-1 peptide fused to its C-terminus. Here, we describe the expression, purification and crystallization processes of this novel construct and report two new structures of PXR_LBD_ that were obtained thanks to this strategy.

## Abstract 2

A versatile construct was designed to improve the purification yield and facilitate structural studies of the ligand-binding domain of the pregnane X receptor.

## 1. Introduction

The human pregnane X receptor (PXR, NR1I2) is a transcription factor belonging to the nuclear receptor (NR) family. PXR has the classical modular structure of NRs, consisting mainly of an N-terminal DNA-binding domain (DBD) and a C-terminal ligand-binding domain (LBD) linked by a hinge region. The general molecular mechanism of NR is as follows: upon ligand binding, changes occur on the receptor surfaces, which represent interaction sites for a wide range of transcriptional coactivators and corepressors (for example the steroid receptor coactivator-1, SRC-1). To date, 66 structures of the PXR_LBD_ have been reported, showing complexes with a diverse range of molecules including drugs (Schneider *et al.*, 2022 ▸; Chrencik *et al.*, 2005 ▸; Lin *et al.*, 2023 ▸), environmental pollutants (Delfosse *et al.*, 2015 ▸, 2021 ▸) and natural compounds (Bartolini *et al.*, 2020 ▸; Fan *et al.*, 2023 ▸). Indeed, the PXR_LBD_ contains a large and versatile ligand-binding pocket (LBP), which enables it to bind a range of molecules with different structures and chemical properties, thereby fulfilling its biological functions (Delfosse *et al.*, 2021 ▸; Hall *et al.*, 2021 ▸; Buchman *et al.*, 2018 ▸). PXR is traditionally regarded as a xenobiotic receptor, and as such it regulates the transcription of metabolizing enzymes and drug transporters, thereby facilitating the removal of xenobiotics from the body (Gee *et al.*, 2024 ▸; Rakateli *et al.*, 2023 ▸; Willson & Kliewer, 2002 ▸). Consequently, PXR plays a role in the early stages of drug metabolism, which can result in adverse effects such as drug–drug interactions, resistance to anticancer therapies or the accumulation of toxic metabolites (Rao *et al.*, 2023 ▸; Feng *et al.*, 2018 ▸; Planque *et al.*, 2016 ▸; Yu *et al.*, 2022 ▸). However, an increasing body of evidence shows that PXR has a broader range of actions, including roles in inflammatory processes and in regulating lipid and glucose metabolism (Rakateli *et al.*, 2023 ▸; Gee *et al.*, 2024 ▸; Lv *et al.*, 2022 ▸; Wang *et al.*, 2022 ▸; Bautista-Olivier & Elizondo, 2022 ▸). Therefore, this receptor is of great interest to the pharmaceutical industry, with the aim of enhancing the efficacy of specific drug treatments and developing novel therapies. Moreover, PXR constitutes a significant target for environmental compounds, making it a subject of interest for the chemical industry with regard to its environmental and public health relevance (Delfosse *et al.*, 2015 ▸, 2021 ▸; Toporova & Balaguer, 2020 ▸).

In the Protein Data Bank, crystal structures of the PXR_LBD_ are mainly reported in two different crystal forms correlated with the construct used for protein production and crystallization (Table 1 ▸). The first form has symmetry consistent with space group *P*2_1_2_1_2_1_, comprising two molecules per asymmetric unit. The LBD of PXR is crystallized in the presence of a peptide that mimics the SRC-1 coactivator. This peptide serves to enhance the stability of PXR throughout the production and purification processes. Two strategies have been established for the introduction of SRC-1. The first involves co-expressing the PXR_LBD_ and a fragment of SRC-1 of about 80 residues (623–710), followed by the addition of an excess of a synthetic SRC-1 peptide (25 residues, 6 76–700) to ensure the stability of the complex during crystallization (Watkins, Maglich *et al.*, 2003 ▸; Lin *et al.*, 2017 ▸). The second strategy involves fusing the PXR_LBD_ to an SRC-1 peptide (23 residues, 678–700) at its C-terminus (Wang *et al.*, 2008 ▸; Garcia-Maldonado *et al.*, 2024 ▸). The second crystal form has the symmetry of space group *P*4_3_2_1_2 and contains only one molecule per asymmetric unit. In this form, the PXR_LBD_ crystallizes alone while the protein is co-expressed, as described previously with the SRC-1 fragment, which is partially lost during purification (Watkins *et al.*, 2001 ▸; Huber *et al.*, 2023 ▸). During our structural studies, we used the co-expressed version for a long time to obtain crystals in space group *P*4_3_2_1_2, but we were confronted with reproducibility problems in terms of purification yield (Delfosse *et al.*, 2015 ▸, 2021 ▸; Schneider *et al.*, 2022 ▸). We have also used a construct based on the so-called tethered PXR_LBD_-SRC-1, which allows easier purification and crystallizes in the *P*2_1_2_1_2_1_ crystal form, but this construct has the disadvantage of retaining SRC-1, which is not always necessary and may even prevent certain biophysical assays (for example receptor–coregulator interaction studies). To overcome this problem, we designed a new PXR_LBD_-Thb-SRC-1 fusion construct that combines the advantage of having good stability and solubility during purification with the possibility of crystallizing the PXR_LBD_ with or without SRC-1 thanks to the introduction of a thrombin cleavage site between the PXR_LBD_ and SRC-1 parts. This construct was used to crystallize the PXR_LBD_ in both crystal forms with its reference ligand SR12813, as well as with two new ligands in the *P*4_3_2_1_2 form. The protocols for the purification and crystallization of these four structures are reported here.

## 2. Materials and methods

### 2.1. Protein production and purification

#### 2.1.1. Cloning

**Figure 1:**
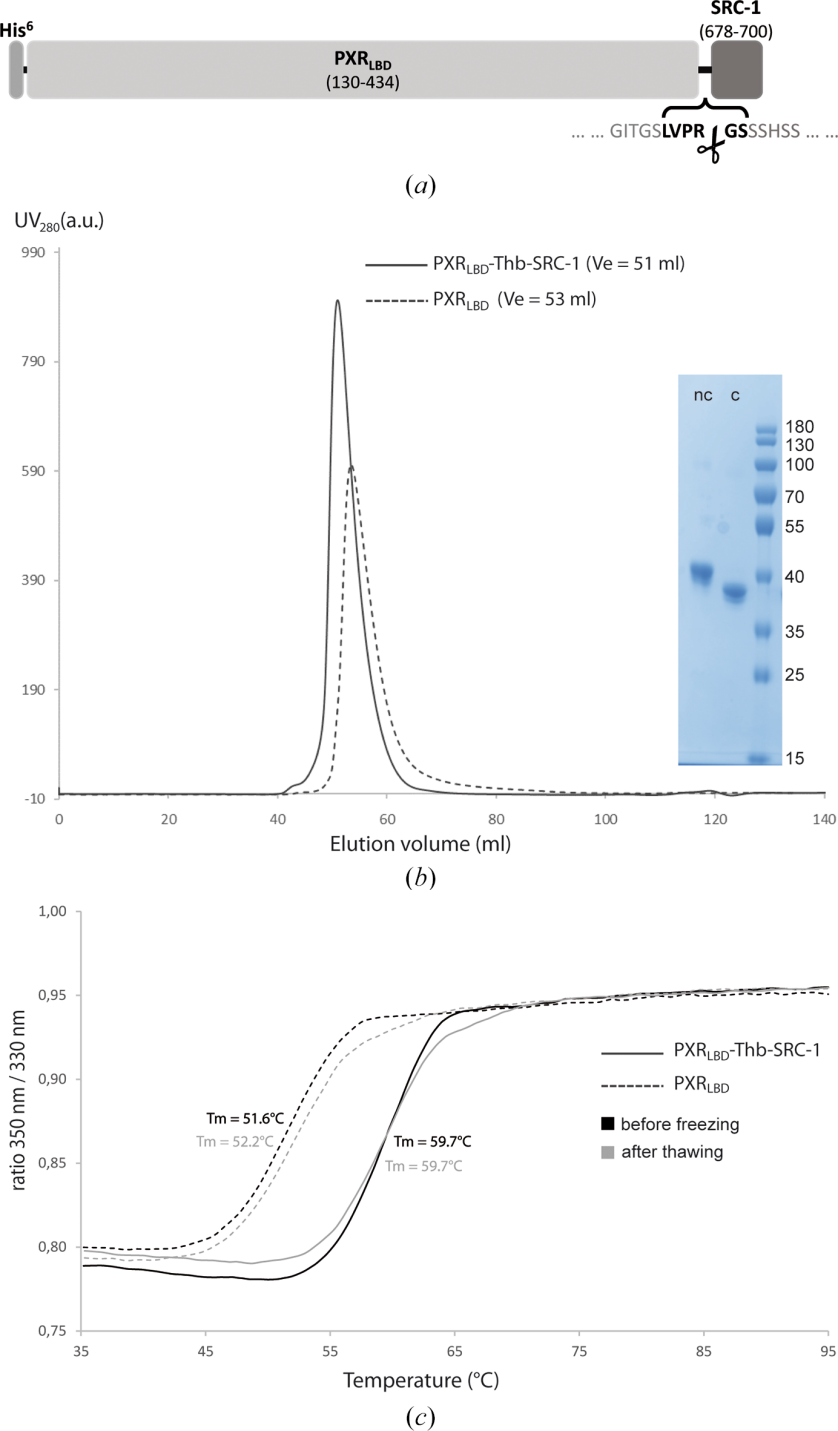
A two-in-one construct that provides a large quantity of pure PXR_LBD_ with or without SRC-1. (*a*) Schematic representation of the primary sequence of the cleavable PXR_LBD_-Thb-SRC-1 construct. (*b*) Gel-filtration elution profiles for the PXR_LBD_-Thb-SRC-1 (solid line) and PXR_LBD_ (dashed line) proteins. The SDS–PAGE inset shows the PXR_LBD_ samples before (lane nc) and after (lane c) thrombin cleavage. The ladder is labelled in kDa. (*c*) Thermal unfolding profiles of the PXR_LBD_-Thb-SRC-1 (solid line) and PXR_LBD_ (dashed line) proteins before freezing (black) and after thawing (gray). For the latter, results are shown for samples that were thawed 18 months after purification.

We designed a DNA fragment containing the coding region for the PXR_LBD_ (residues 130–434, UniProt entry O75469) fused, in its C-terminal part, to the coding region for the SRC-1 NR box II (residues 678–700, UniProt entry Q15788). In the hinge sequence located between the PXR_LBD_ and the SRC-1, we inserted a sequence coding for a thrombin cleavage site (LVPR/GS; Fig. 1 ▸*a*; Table 2 ▸). In addition, the 5′ and 3′ extremities contained 15 base pairs required for In-Fusion cloning (Clontech), including the restriction sites NdeI and HindIII, respectively. The chimeric sequence was synthesized and cloned into the pET-11a expression vector (Novagen) by following the instructions for the In-Fusion cloning protocol in such a way that the final construct contains an N-terminal His_6_ tag (see the supporting information). The construct was sequenced to confirm that no mutations had occurred during the cloning procedures. The resulting protein will subsequently be referred to as PXR_LBD_-Thb-SRC-1.

#### 2.1.2. Production

The plasmid was transformed into an *Escherichia coli* BL21(DE3) expression cell line and selected using ampicillin. Three colonies were used to inoculate a 500 ml flask containing 200 ml LB broth supplemented with 100 µg ml^−1^ ampicillin. The culture was grown overnight at 37°C. This preculture was used to inoculate two 2 l flasks containing 500 ml ZYM medium supplemented with 100 µg ml^−1^ ampicillin at an OD_600_ of 0.15 (see the supporting information). The culture was incubated for 4 h at 37°C and then overnight at 25°C. The cells were harvested by centrifugation at 6000*g* for 20 min. The resulting pellets were collected and stored at −40°C.

#### 2.1.3. Purification

All steps were performed at 4°C, and aliquots were collected during the process to control for the presence and the purity of the proteins by SDS–PAGE. The pellets resulting from 2 × 500 ml culture were thawed on ice and homogenized using an Ultra-Turrax T-25 in 200 ml lysis buffer (20 m*M* Tris–HCl, 150 m*M* NaCl, 5% glycerol pH 8.0 at 4°C) supplemented with 200 µl of a 10 mg ml^−1^ lysozyme solution and two protease-inhibitor cocktail tablets (Complete EDTA-free, Roche). After 20 min of gentle stirring, the lysate was further processed by sonication with a Sonic Ruptor 4000 (power 40%, pulser 50%, 4 × 2 min) and then centrifugated for 30 min at 18 000*g*. The supernatant was manually and sequentially filtered using 5 and 0.45 µm filters. The following steps were performed with the help of an ÄKTApure system (Cytiva) located in a cold room. The cleared lysate was loaded onto a 5 ml Ni–NTA Superflow Cartridge (Qiagen) equilibrated with lysis buffer supplemented with 20 m*M* imidazole, *i.e.* 4% elution buffer (20 m*M* Tris–HCl, 150 m*M* NaCl, 500 m*M* imidazole, 5% glycerol, pH 8.0 at 4°C) at a flow rate of 1.5 ml min^−1^. In order to eliminate nonspecifically bound proteins, the column was washed with 30 column volumes (CV) of 4% elution buffer (final concentration of 20 m*M* imidazole) and then with 10 CV of 10% elution buffer (final concentration of 50 m*M* imidazole) at a flow rate of 3 ml min^−1^. The PXR_LBD_-Thb-SRC-1 fusion protein was eluted with 5 CV of 35% elution buffer (final concentration of 175 m*M* imidazole) at a flow rate of 3 ml min^−1^. The column was further washed with 10 CV of 100% elution buffer (final concentration of 500 m*M* imidazole) (Supplementary Fig. S1*a*). Following the affinity-chromatography step, the pooled PXR_LBD_-Thb-SRC-1-containing fractions were treated differently depending on whether or not it was desired to retain the SRC-1 peptide. In the first case, the eluted protein was concentrated with an Amicon Ultra-15 concentrator device (molecular-weight cutoff of 10 000, Millipore; several 15 min rounds of centrifugation at 3500*g* at 4°C) to reach a final volume of about 1.5 ml. The concentrated PXR_LBD_-Thb-SRC-1 sample was then centrifugated for 10 min at 10 000*g* to remove potential precipitates and applied onto a HiLoad 16/60 Superdex 75 prep-grade gel-filtration column (Cytiva) equilibrated with protein storage buffer (20 m*M* Tris–HCl, 250 m*M* NaCl, 1 m*M* DTT, 5% glycerol, pH 8.0 adjusted at 4°C). The PXR_LBD_-Thb-SRC-1-containing fractions (Fig. 1 ▸*b* and Supplementary Fig. S1*b*) were pooled and concentrated to 10 mg ml^−1^ before crystallization experiments or storage at −40 or −80°C. In the case in which removal of the SRC-1 peptide was desired, it was cleaved with thrombin. The protein eluted from the nickel-affinity column was concentrated with an Amicon Ultra-15 concentrator device (molecular-weight cutoff of 10 000, Millipore; several 15 min rounds of centrifugation at 3500*g* at 4°C) to reach a final concentration of about 2–3 mg ml^−1^. Thrombin (catalogue No. T7513, Sigma–Aldrich) was added to one unit per milligram of PXR_LBD_-Thb-SRC-1 (*i.e.* 0.5 µg per milligram of PXR_LBD_-Thb-SRC-1) and the sample was then dialyzed overnight against 1 l protein storage buffer at 4°C. The cleaved PXR_LBD_ was further concentrated to reach a final volume of about 1.5 ml, centrifugated for 10 min at 10 000*g* to remove potential precipitates and then applied onto a HiLoad 16/60 Superdex 75 prep-grade gel-filtration column (Cytiva) equilibrated with protein storage buffer (20 m*M* Tris–HCl, 250 m*M* NaCl, 1 m*M* DTT, 5% glycerol, pH 8.0 adjusted at 4°C). The PXR_LBD_-containing fractions (Fig. 1*b* and Supplementary Fig. S1*b*) were pooled and concentrated to 4 mg ml^−1^ before crystallization experiments or storage at −40 or −80°C.

### 2.2. Thermal denaturation

To ensure the quality of the protein samples and the consistency of the purification batches, the melting temperature was systematically controlled after purification and before crystallization trials using nano-differential scanning fluorimetry (nanoDSF). 10 µl of PXR_LBD_ or PXR_LBD_-Thb-SRC-1 at 1 mg ml^−1^ was run on a Tycho NT.6 system in Tycho capillaries (NanoTemper Technologies; Fig. 1 ▸*c*). Samples were heated progressively from 35 to 95°C at a defined rate of 30°C min^−1^ and intrinsic protein fluorescence from tryptophan and tyrosine residues was recorded at 330 and 350 nm throughout the run. The data were automatically analyzed by the system software to calculate the inflection temperature (*T*_m_), which indicates the temperature at which the proteins unfold.

### 2.3. Crystallization

**Figure 2:**
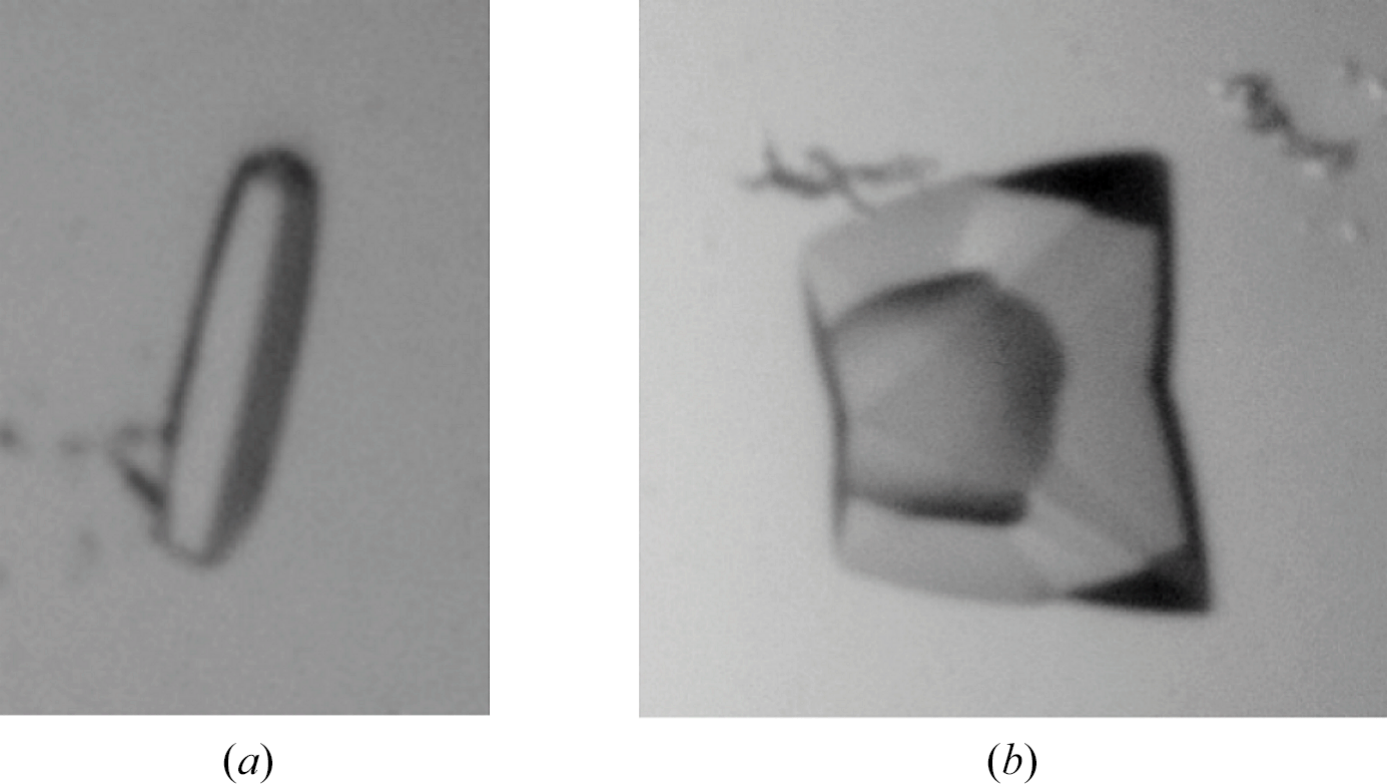
Two crystal forms, two morphologies. (*a*) Crystal of PXR_LBD_-Thb-SRC-1 grown at 4°C in space group *P*2_1_2_1_2_1_. (*b*) Crystal of PXR_LBD_ grown at 20°C in space group *P*4_3_2_1_2.

Crystals of the SR12813–PXR_LBD_-Thb-SRC-1 complex were obtained by co-crystallization (Table 3 ▸; Fig. 2 ▸*a*). Co-crystals of PXR_LBD_ in complex with SR12813, JMV6995 or JMV7035 were obtained similarly (Table 3 ▸; Fig. 2 ▸*b*). Crystallization conditions were designed based on the literature. Ligands were stored and used in 100% DMSO at 10 m*M*. Crystals appeared in 24–72 h and, to avoid difficulties due to the formation of a skin around the crystallization drop, we harvested and flash-cooled the crystals within three days. No cryoprotectant was needed due to the presence of alcohols in the crystallization buffers.

### 2.4. Structure determination

#### 2.4.1. Data collection and processing

Data were processed and scaled with *XDS* and *XSCALE* (Kabsch, 2010*a* ▸,*b* ▸). Data-collection and processing statistics are reported in Table 4 ▸.

#### 2.4.2. Structure solution and refinement

The structures were determined by molecular replacement (using PDB entry 1ilg as a search model; Watkins *et al.*, 2001 ▸) using *Phenix* (Liebschner *et al.*, 2019 ▸) and refined using *Phenix* and *Coot* (Emsley *et al.*, 2010 ▸). Refinement statistics are summarized in Table 5 ▸. Figures were prepared with *PyMOL* (https://pymol.org/).

## 3. Results and discussion

The first structures of the PXR_LBD_ were elucidated by the group of M. R. Redinbo in 2001 in the apo form and in complex with the pharmaceutical compound SR12813, and without the SRC-1 coactivator peptide (Watkins *et al.*, 2001 ▸). Currently, approximately 60 additional structures have been reported, and the PXR_LBD_ remains a subject of interest in the fields of drug design and environmental science. These structures were obtained from crystals with the symmetry of space groups *P*2_1_2_1_2_1_ or *P*4_3_2_1_2 depending on the presence or absence of the SRC-1 peptide, respectively. In both cases, the PXR_LBD_ is produced and purified in the presence of an SRC-1 peptide, which ensures its solubility and stability. The peptide was initially introduced into the cultures of the PXR_LBD_ via a co-expression system comprising two plasmids (Watkins *et al.*, 2001 ▸). Two alternative strategies have since emerged that facilitate the production of the PXR_LBD_: (i) the so-called ‘tethered construct’, resulting in a PXR_LBD_-SRC-1 fusion protein (Wang *et al.*, 2008 ▸), and (ii) the ‘Duet construct’, which avoids modification of the PXR_LBD_ by using a dual promoter expression vector (Lin *et al.*, 2023 ▸). Although both methods guarantee equimolarity during production, the second method presents the disadvantage of requiring the addition of a synthetic peptide during the purification process to keep the protein stable. As part of our structural studies, we initially used the co-expression system kindly provided by M. R. Redinbo without incorporating a synthetic peptide throughout the purification and crystallization processes. Our objective was to examine the binding mode of environmental ligands on the PXR_LBD_ without the SRC-1 peptide that reinforces the active conformation. This approach ensured that the orientation of the ligands and the conformation of the protein were not biased by this factor. The difficulties encountered in obtaining a sufficient quantity of pure protein in a reproducible manner for use in crystallization trials prompted us to redesign the tethered construct. The novelty of this construct lies in the presence of a thrombin cleavage site in the junction region between the PXR_LBD_ and SRC-1 parts (Fig. 1 ▸*a*), where the –GGSGG– sequence has been replaced by –LVPRGS–. This simple construct ensures the equimolar presence of the coactivator, which is required for better solubilization and stabilization of the PXR_LBD_ throughout the production and purification process, without adding a synthetic peptide. It also allows removal of SRC-1 at the final step, when the protein is nearly pure and less prone to precipitation. Thus, by overproducing the protein in ZYM medium, we can obtain ∼10 mg of pure PXR_LBD_ or ∼30 mg of pure PXR_LBD_-Thb-SRC-1 per litre of culture, respectively, which are stable over time when frozen (Figs. 1 ▸*b* and 1 ▸*c*). The difference in purification yield between the two forms is directly linked to the intrinsic stability of the LBD and has previously been reported (Wang *et al.*, 2008 ▸). The SRC-1 peptide allows stabilization of the PXR_LBD_, thus facilitating its concentration during the different stages of purification. Precipitates were observed during the concentration of the PXR_LBD_ before and after the gel-filtration step, which explains the lower yield obtained for this form. This behavior of the proteins is confirmed by their respective melting temperatures, which differ by about 8°C: 51–52°C for the PXR_LBD_ and 59–60°C for the PXR_LBD_-Thb-SRC-1 (Fig. 1 ▸*c*).

**Figure 3:**
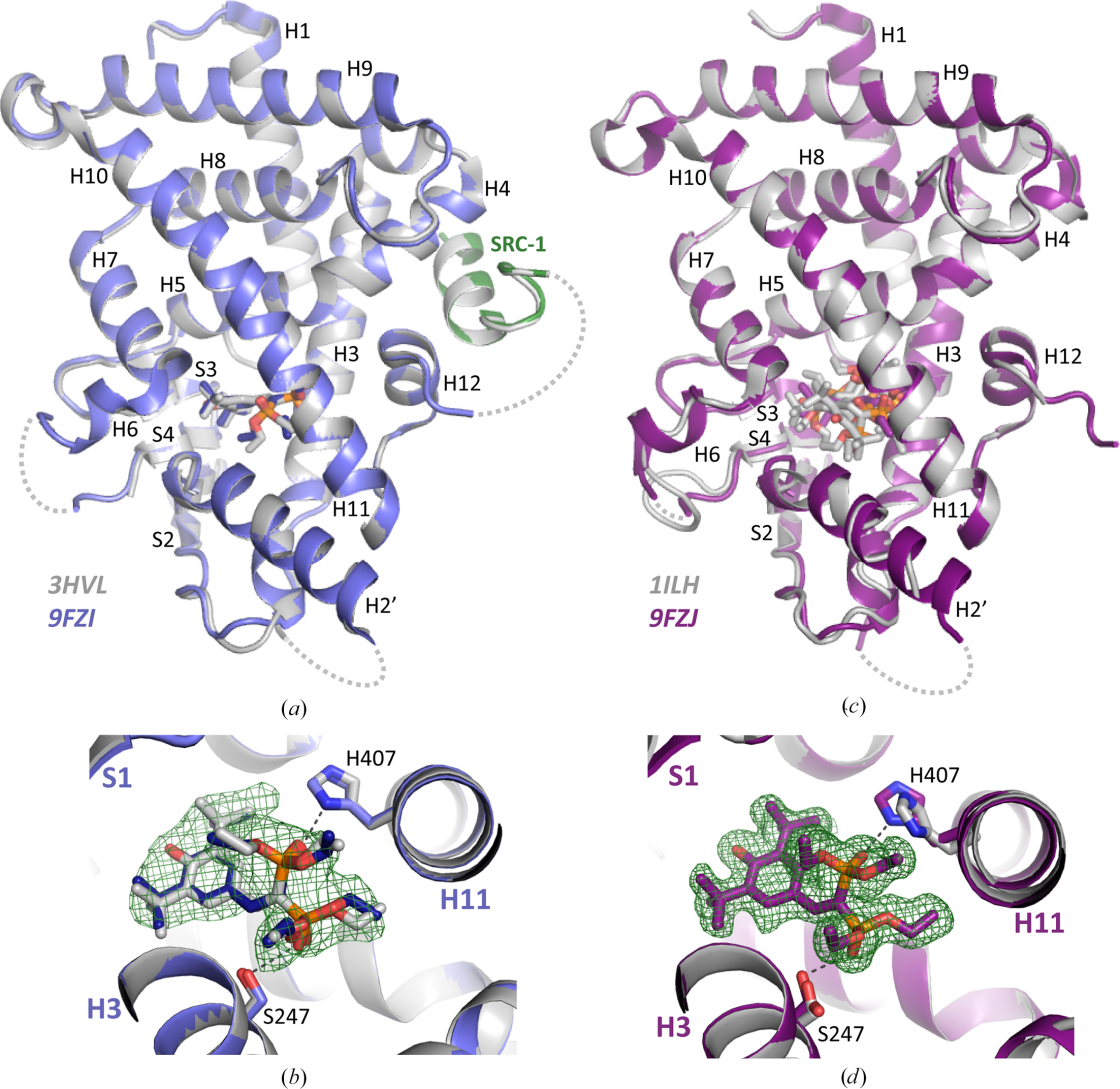
The novel cleavable linker does not affect the structural configuration of the PXR_LBD_. (*a*) Superimposition of a known structure (PDB entry 3hvl; gray) and the new structure (PDB entry 9fzi; blue) of PXR_LBD_-SRC-1 in complex with SR12813 (r.m.s.d. of 0.9 Å over 275 C^α^ atoms). The SRC-1 peptide moiety is represented in green. (*b*) A close-up view of the ligand-binding pockets in the PXR_LBD_-SRC-1 structures. The *F*_o_ − *F*_c_ simulated-annealing omit map is presented at an r.m.s.d. of 2 for PDB entry 9fzi (2.7 Å resolution). (*c*) Superimposition of the known structure (PDB entry 1ilh; gray) and new structure (PDB entry 9fzj; purple) of PXR_LBD_ in complex with SR12813 (r.m.s.d. of 1.2 Å over 241 C^α^ atoms). In PDB entry 1ilh, SR12813 was modeled in three different orientations, unlike PDB entry 9fzj, where it present in one. (*d*) A close-up view of the ligand-binding pockets in the PXR_LBD_ structures. The ligand of PDB entry 1ilh is not shown for clarity. The *F*_o_ − *F*_c_ simulated-annealing omit map is presented at an r.m.s.d. of 2 for PDB entry 9fzj (1.6 Å resolution). Oxygen, red; nitrogen, blue; phosphorus, orange. Black dashed lines show hydrogen bonds. Gray dashed lines represent flexible regions that are not visible in the crystal structures.

In order to assess the usefulness of the new construct and to verify that the new linker does not affect the process of crystallization, we determined the structure of the PXR_LBD_ in complex with the reference ligand SR12813 with and without the SRC-1 peptide. The two forms were crystallized under the respective conditions and in the space groups (Table 3 ▸) previously described in the literature. The crystals of the SR12813–PXR_LBD_-Thb-SRC-1 complex, which have the symmetry of space group *P*2_1_2_1_2_1_ with two molecules per asymmetric unit, were grown at 4°C in MPD, whereas those of the SR12813–PXR_LBD_ complex have the symmetry of space group *P*4_3_2_1_2, with one molecule per asymmetric unit, and were obtained at room temperature in 2-propanol. The structures were determined by molecular replacement using the apo form of the PXR_LBD_ as a search model (PDB entry 1ilg; Watkins *et al.*, 2001 ▸). As expected, the SR12813–PXR_LBD_-Thb-SRC-1 complex structure is very similar to the structure of the tethered version (PDB entry 3hvl; Wang *et al.*, 2008 ▸), with an r.s.m.d. of 0.9 Å over 275 matching C^α^ atoms (LSQ calculation). As observed in the initial structure (Wang *et al.*, 2008 ▸), the linker between the PXR_LBD_ and the SRC-1 parts, which is probably disordered, is not visible (Fig. 3 ▸*a*). Only the SRC-1 peptide region interacting with the PXR_LBD_ is well defined. Similarly, the region preceding H2′ (more or less including residues 176–209), which is absent in all of the PXR_LBD_ and PXR_LBD_-SRC-1 structures listed in Table 1 ▸, is no more clearly defined in the new model (Fig. 3 ▸*a*). A closer examination of the LBP revealed that the SR12813 ligand maintains its original orientation and specifically interacts with the protein as described previously. These interactions involve the formation of hydrogen bonds to the side chains of residues Ser247 and His407 (Fig. 3 ▸*b*). In the same way, the SR12813–PXR_LBD_ complex structure superimposed perfectly on the original structure (PDB entry 1ilh; Watkins *et al.*, 2001 ▸), with an r.s.m.d. of 1.2 Å over 241 matching C^α^ atoms (LSQ calculation) (Fig. 3 ▸*c*). The only significant difference between the two structures lies in the presence of a single orientation for SR12813, in contrast to the initial structure in which three orientations were modeled for this ligand (Watkins *et al.*, 2001 ▸). As a consequence, minor differences are observed for some residue side chains within the LBP. The very well defined *F*_o_ − *F*_c_ omit map clearly shows that in the new structure the ligand adopts the orientation that is also observed in the SR12813–PXR_LBD_-SRC-1 complex structure (Fig. 3 ▸*d*). These different observations may be attributed to the rather different resolutions at which the structures were determined: 2.76 Å (PDB entry 1ilh; Watkins *et al.*, 2001 ▸) and 1.60 Å (PDB entry 9fzj).

**Figure 4:**
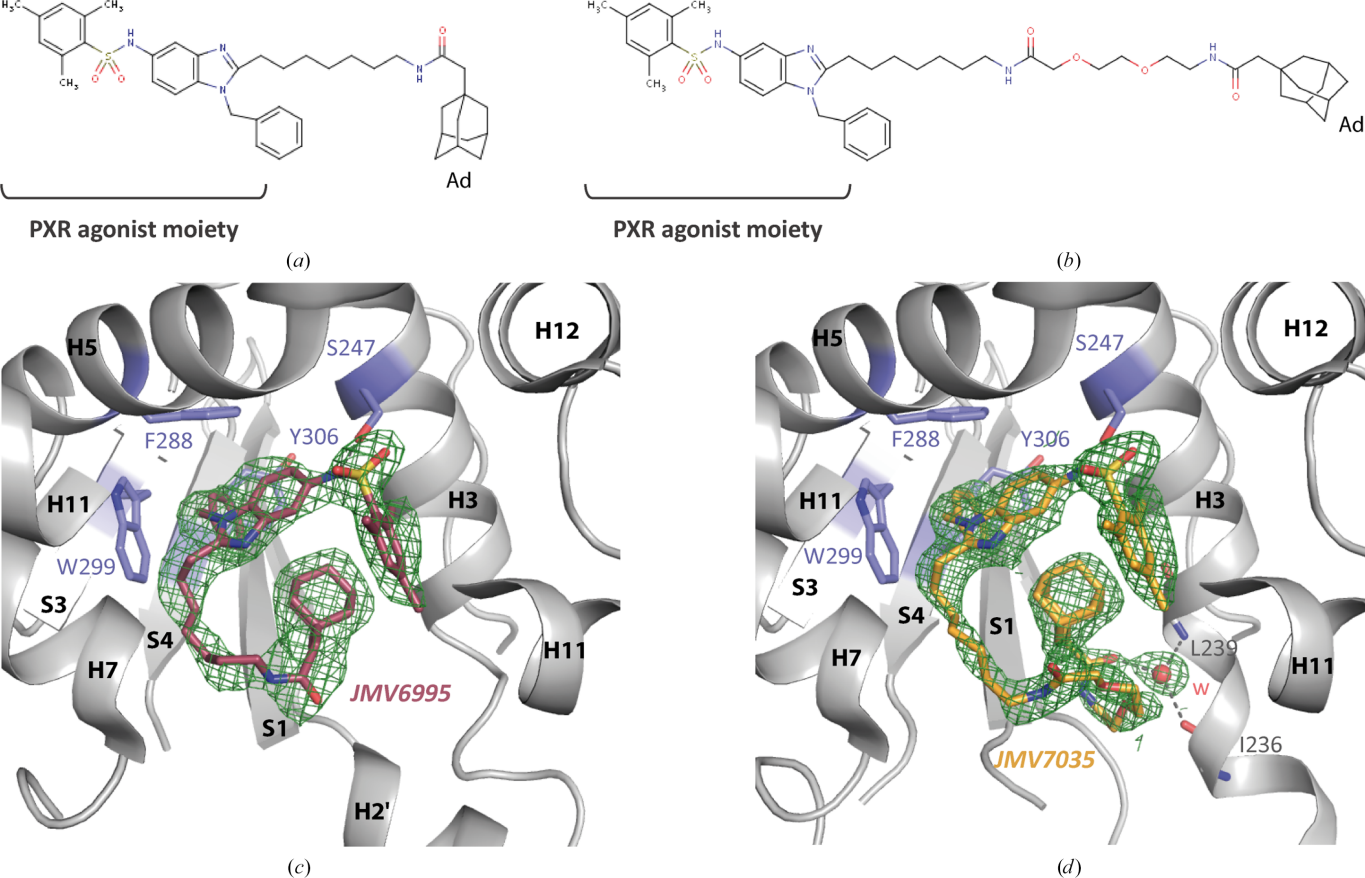
New structures of the PXR_LBD_ in the absence of the SRC-1 peptide. 1D chemical structures of (*a*) JMV6995 and (*b*) JMV7035 (Ad, adamantane). Close-up views of the ligand-binding pocket of the PXR_LBD_ in complex with (*c*) JMV6995 (2.5 Å resolution) and (*d*) JMV7035 (2.0 Å resolution). The 2*mF*_o_ − *F*_c_ simulated-annealing omit maps are represented at an r.m.s.d. of 1. For clarity, the H11 helix at the front has been truncated. Oxygen, red; nitrogen, blue; sulfur, yellow; w, water. Black dashed lines show hydrogen bonds.

Following the successful implementation of the new cleavable construct, we have determined the structures of novel complexes with the PXR_LBD_. This was performed as part of a project aimed at developing a PROTAC (PROteolysis TArgeting Chimera) ligand targeting PXR (Bansard *et al.*, 2024 ▸). PROTACs are bifunctional molecules that work by bridging a specific protein to an E3 ligase, forming a ternary complex. In this way, the PROTAC targets the protein of interest for degradation by hijacking the E3 ligase and the ubiquitin–proteasome system (Pettersson & Crews, 2019 ▸). In this context, we have also tested the hydrophobic tagging technology (HyT-technology), whereby proteins become unstable and are subsequently degraded by the proteasome following the addition of a hydrophobic group (for example adamantane) to their surface (Wang *et al.*, 2020 ▸; He *et al.*, 2023 ▸). Two new synthetic compounds, named JMV6995 and JMV7035 (Figs. 4 ▸*a* and 4 ▸*b*), have been developed for this purpose. They contain an adamantane moiety linked by a spacer arm to a PXR agonist-based scaffold (Benod *et al.*, 2008 ▸). The difference between the two lies in the size and the chemical nature of the linker, with JMV7035 containing an additional PEG spacer. JMV6995 and JMV7035 were co-crystallized with the PXR_LBD_ and were unambiguously positioned in the electron density of the resulting structures determined at 2.50 and 2.00 Å resolution, respectively (Fig. 4 ▸). This makes JMV7035 the largest ligand to be crystallized with the PXR_LBD_ to date (molecular weight 840.12 g mol^−1^). Surprisingly, the structures show that the adamantane groups of these compounds did not reach the surface of the PXR_LBD_, as expected, but were fully internalized into the LBP (Fig. 4 ▸). JMV6995 and JMV7035 mainly interact with the PXR_LBD_ through van der Waals and hydrophobic contacts. Only one hydrogen bond is observed to the side chain of Ser247. In both cases, the mesityl sulfonamide group of the PXR agonist moiety is situated between helices H3 and H11, in close proximity to H12 (Figs. 4 ▸*c* and 4 ▸*d*). On the other side of the pocket, the benzyl group is embedded within the aromatic π-trap, which is formed by Phe288, Trp299 and Tyr306 (Delfosse *et al.*, 2021 ▸). The adamantane group is able to fit into the hydrophobic environment created by the aforementioned mesityl sulfonamide and the benzyl groups, with the linker making its way into the rest of the pocket. In the JMV6995 complex, the positioning of the linker has an impact on the stability of loops H2′–S1 and S4–H6, and as a result residues 207–209 and 310–317 could not be modeled (Fig. 4 ▸*c*). With regard to JMV7035, the linker initially follows a similar path and the adamantane group is perfectly superimposed on that of JMV6995 (Figs. 4 ▸*c* and 4 ▸*d*). Furthermore, the positioning of the PEG chain between these two components results in destabilization of the H2′ helix, which is no longer defined by the electron density. Accordingly, the region including residues 178–207 was not modeled. Finally, the carbonyl group of the linker forms a hydrogen bond to a water molecule in the LBP, which in turn interacts via hydrogen bonds to the Ile236 and Leu239 backbones (Fig. 4 ▸*d*). The unobserved regions of these complex structures have been previously described as highly dynamic and, depending on the size of the ligand, could be significantly affected. This is particularly the case for rifampicin, the structure of which showed no clear electron density for residues 178–209, 229–235 and 310–317 (Chrencik *et al.*, 2005 ▸; Garcia-Maldonado *et al.*, 2024 ▸). Thus, the JMV6995 and JMV7035 complex structures, in which the ligands fold on themselves, once again demonstrate the extraordinary adaptability of the LBP of this receptor.

In summary, we have designed a novel construct for the production and purification of high yields of the PXR_LBD_ in fusion with a cleavable SRC-1-derived coactivator peptide that can be removed at will. The choice of one or the other of the resulting proteins will be determined by the studies that are to subsequently be performed. For example, the PXR_LBD_-Thb-SRC-1 fusion is well suited for use in isothermal titration calorimetry (ITC; Delfosse *et al.*, 2015 ▸, 2021 ▸) or hydrogen–deuterium exchange mass spectrometry (HDX-MS) experiments that require a stable sample over time. In addition, the fusion protein is suitable for ligand-interaction studies, whereas the cleaved form must be used for coregulator interaction experiments. In the context of crystallographic studies, we prefer to use the PXR_LBD_, for which we were able to determine two new complex structures. This versatile construct is likely to be of interest to other academic and pharmaceutical laboratories interested in structural studies of PXR, particularly for drug-design purposes.

## Supplementary Material

PDB reference: human pregnane X receptor ligand-binding domain, complex with compound JMV7035, 9fzg

PDB reference: complex with compound JMV6995, 9fzh

PDB reference: fusion with an SRC1 co-activator peptide, 9fzi

PDB reference: complex with SR12813 (*P*4_3_2_1_2 form), 9fzj

Supplementary Protocols and Supplementary Figure. DOI: 10.1107/S2053230X2500069X/rf5045sup1.pdf

## Figures and Tables

**Table 1:** PXR_LBD_ structures by crystal form

PXR_LBD_-SRC-1 (*P*2_1_2_1_2_1_)	1nrl (Watkins, Davis-Searles *et al.*, 2003 ▸); 2o91 (Xue *et al.*, 2007 ▸); 3ctb, 3hvl (Wang *et al.*, 2008 ▸); 5a86 (Hennessy *et al.*, 2015 ▸); 5x0r (Lin *et al.*, 2017 ▸); 6bns (Gong *et al.*, 2018 ▸); 6dup (Vaz *et al.*, 2018 ▸); 6s41 (Focken *et al.*, 2019 ▸); 6hty (Werner *et al.*, 2019 ▸); 6nx1 (Duan *et al.*, 2019 ▸); 6p2b (Bartolini *et al.*, 2020 ▸); 6xp9 (Sivaprakasam *et al.*, 2020 ▸); 6tfi (Hillisch *et al.*, 2020 ▸); 8eqz (Lin *et al.*, 2023 ▸); 7yfk (Fan *et al.*, 2023 ▸); 8f5y (Huber *et al.*, 2024 ▸); 8svn, 8svo, 8svp, 8svq, 8svr, 8svs, 8svt, 8svu, 8svx (Garcia-Maldonado *et al.*, 2024 ▸)
PXR_LBD_ (*P*4_3_2_1_2)	1ilg, 1ilh (Watkins *et al.*, 2001 ▸); 1m13 (Watkins, Maglich *et al.*, 2003 ▸); 1skx (Chrencik *et al.*, 2005 ▸); 2qnv (D. G. Teotico, J. Bischof & M. R. Redinbo, unpublished work); 3r8d (Cheng & Redinbo, 2011 ▸); 4ny9 (Santella *et al.*, 2014 ▸); 4xhd (Khan *et al.*, 2015 ▸); 4x1f, 4x1g, 4xao (Delfosse *et al.*, 2015 ▸); 6hj2 (Schneider *et al.*, 2022 ▸); 7ax8, 7ax9, 7axa, 7axb, 7axc, 7axd, 7axe, 7axf, 7axg, 7axh, 7axi, 7axj, 7axk, 7axl (Delfosse *et al.*, 2021 ▸); 7n2a, 7rio, 7riu, 7riv (Ramanjulu *et al.*, 2021 ▸); 8e3n, 8fpe (Lin *et al.*, 2023 ▸); 8szv (Huber *et al.*, 2023 ▸); 8cct, 8cf9, 8ch8 (C. Carivenc, Q. Derosa, M. Grimaldi, A. Boulahtouf, P. Balaguer & W. Bourguet, unpublished work)

**Table 2:** Macromolecule-production information

Source organism	*Homo sapiens*
DNA source	Synthetic gene (IDT)
Expression vector	pET-11a
Plasmid construction method	In-Fusion cloning (Clontech)
Expression host	*Escherichia coli* BL21(DE3)
Expression details	Autoinduction medium (see the supporting information for details)
Complete amino-acid sequence of the protein produced (PXR_LBD_-Thb-SRC-1)†	MKKG**HHHHHH**GSERTGTQPLGVQGLTEEQRMMIRELMDAQMKTFDTTFSHFKNFRLPGVLSSGCELPESLQAPSREEAAKWSQVRKDLCSLKVSLQLRGEDGSVWNYKPPADSGGKEIFSLLPHMADMSTYMFKGIISFAKVISYFRDLPIEDQISLLKGAAFELCQLRFNTVFNAETGTWECGRLSYCLEDTAGGFQQLLLEPMLKFHYMLKKLQLHEEEYVLMQAISLFSPDRPGVLQHRVVDQLQEQFAITLKSYIECNRPQPAHRFLFLKIMAMLTELRSINAQHTQRLLRIQDIHPFATPLMQELFGITGS**LVPRGS**SSHSSLTERHKILHRLLQEGSPS
Amino-acid sequence of the protein after thrombin cleavage (PXR_LBD_)†	MKKG**HHHHHH**GSERTGTQPLGVQGLTEEQRMMIRELMDAQMKTFDTTFSHFKNFRLPGVLSSGCELPESLQAPSREEAAKWSQVRKDLCSLKVSLQLRGEDGSVWNYKPPADSGGKEIFSLLPHMADMSTYMFKGIISFAKVISYFRDLPIEDQISLLKGAAFELCQLRFNTVFNAETGTWECGRLSYCLEDTAGGFQQLLLEPMLKFHYMLKKLQLHEEEYVLMQAISLFSPDRPGVLQHRVVDQLQEQFAITLKSYIECNRPQPAHRFLFLKIMAMLTELRSINAQHTQRLLRIQDIHPFATPLMQELFGITGS**LVPR**

† **HHHHHH**, His_6_ tag; **LVPRGS**, thrombin cleavage site.

**Table 3:** Crystallization conditions

	PXR_LBD_-Thb-SRC-1 (*P*2_1_2_1_2_1_)	PXR_LBD_ (*P*4_3_2_1_2)
Method	Hanging-drop vapor diffusion (manual)	Hanging-drop vapor diffusion (manual)
Plate type	EasyXtal 15-Well Tool + EasyXtal DropGuard Supports (Molecular Dimensions)	EasyXtal 15-Well Tool + EasyXtal DropGuard Supports (Molecular Dimensions)
Temperature (K)	277	293
Protein concentration (mg ml^−1^/µ*M*)	9–11/228–280	3–5/82–137
Protein buffer	50 m*M* Tris pH 8.0, 250 m*M* NaCl, 5%(*v*/*v*) glycerol, 1 m*M* DTT	50 m*M* Tris pH 8.0, 250 m*M* NaCl, 5%(*v*/*v*) glycerol, 1 m*M* DTT
Compound concentration	Threefold molar excess of protein	Threefold molar excess of protein
Protein–ligand incubation time	1 h at 4°C	1 h at 4°C
Reservoir solution	100 m*M* imidazole pH 7.0–7.4, 25–30%(*v*/*v*) MPD	75 m*M* imidazole pH 7.0–7.4, 8–14%(*v*/*v*) 2-propanol
Volume and ratio of drop	1 µl + 1 µl	1 µl + 1 µl
Volume of reservoir (µl)	500	500

**Table 4:** Data-collection and processing statistics Values in parentheses are for the highest resolution shell.

Protein	PXR_LBD_-Thb-SRC-1	PXR_LBD_	PXR_LBD_	PXR_LBD_
Ligand	SR12813	SR12813	JMV6995	JMV7035
PDB code	9fzi	9fzj	9fzh	9fzg
Diffraction source	ID30A-3, ESRF	ID30A-1, ESRF	ID30A-1, ESRF	ID29, ESRF
Wavelength (Å)	0.9677	0.9660	0.9660	0.9793
Temperature (K)	100	100	100	100
Detector	EIGER 4M	PILATUS3 2M	PILATUS3 2M	PILATUS 6M-F
Distance to detector (mm)	181.09	234.93	236.32	356.64
Total rotation range (°)	180	180	113	125
Rotation per image (°)	0.15	0.20	0.10	0.10
Exposure time per image (s)	0.005	0.100	0.116	0.037
Space group	*P*2_1_2_1_2_1_	*P*4_3_2_1_2	*P*4_3_2_1_2	*P*4_3_2_1_2
*a*, *b*, *c* (Å)	85.42, 89.03, 106.66	91.52, 91.52, 85.70	90.77, 90.77, 87.33	91.04, 91.04, 86.60
α, β, γ (°)	90.00, 90.00, 90.00	90.00, 90.00, 90.00	90.00, 90.00, 90.00	90.00, 90.00, 90.00
Mosaicity (°)	0.20	0.07	0.35	0.14
Resolution (Å)	44.51–2.70 (2.77–2.70)	40.37–1.60 (1.64–1.60)	40.27–2.50 (2.56–2.50)	40.71–2.00 (2.05–2.00)
Total No. of reflections	156162 (12160)	541498 (15290)	114522 (9478)	223611 (16036)
No. of unique reflections	22826 (1689)	48371 (3369)	15251 (1110)	25135 (1814)
Completeness (%)	99.3 (99.8)	99.6 (95.7)	96.4 (100.0)	99.8 (99.9)
Multiplicity	6.8 (7.2)	11.2 (4.5)	7.3 (8.4)	8.9 (8.8)
Mean *I*/σ(*I*)	11.98 (2.70)	32.9 (2.9)	27.1 (4.9)	28.9 (4.6)
*R*_meas_	0.103 (0.581)	0.036 (0.498)	0.051 (0.303)	0.046 (0.534)
CC_1/2_ (%)	99.8 (85.1)	100.0 (83.5)	99.9 (97.0)	100.0 (92.7)
Wilson *B* factor (Å^2^)	56	25	43	33

**Table 5:** Refinement statistics Values in parentheses are for the highest resolution shell.

Protein	PXR_LBD_-Thb-SRC-1	PXR_LBD_	PXR_LBD_	PXR_LBD_
Ligand	SR12813	SR12813	JMV6995	JMV7035
PDB entry	9fzi	9fzj	9fzh	9fzg
Resolution (Å)	44.51–2.70 (2.82–2.70)	40.37–1.60 (1.63–1.60)	40.27–2.50 (2.69–2.50)	40.71–2.00 (2.08–2.00)
No. of reflections	22821 (2820)	48366 (2685)	12658 (2476)	25129 (2729)
*R*_work_/*R*_free_	0.194/0.237 (0.264/0.318)	0.182/0.208 (0.289/0.353)	0.216/0.256 (0.262/0.354)	0.191/0.237 (0.238/0.286)
No. of non-H atoms				
All	4610	2641	2128	2339
Protein	4501	2295	2029	2060
Ligand	66	51	50	80
Water	43	295	49	199
*B* factors (Å^2^)				
Overall	54	32	46	38
Protein	54	31	46	36
Ligand	55	37	52	48
Water	47	43	43	45
R.m.s. deviations				
Bond lengths (Å)	0.009	0.006	0.009	0.008
Bond angles (°)	1.096	0.844	1.086	0.960
Ramachandran plot†				
Favored regions (%)	96.78	98.17	98.04	98.81
Outliers (%)	0	0	0	0

† Calculated with *MolProbity* (Williams *et al.*, 2018 ▸).
