# Supplementary material for: A two-in-one expression construct for biophysical and structural studies of the human pregnane X receptor ligand-binding domain, a pharmaceutical and environmental target
Source: Acta Crystallogr F Struct Biol Commun. 2025 Feb 9;81(Pt 3):85–94. doi: 10.1107/S2053230X2500069X (PMC11866411; doi:10.1107/S2053230X2500069X)
Supplement: Supplementary file 1 [file f-81-00085-sup1.pdf]

STRUCTURAL BIOLOGY
COMMUNICATIONS

Volume 81 (2025)

Supporting information for article:

A two-in-one expression construct for biophysical and structural studies of the human pregnane X receptor ligand-binding domain, a pharmaceutical and environmental target

Coralie Carivenc, Guillaume Laconde, Pauline Blanc, Muriel Amblard, William Bourguet and Vanessa Delfosse

S1. Supplementary protocol

S1.1. Plasmid sequence

```
> [pET11a_PXR-SRC1.xdna - 6501 bp]
aggagatTTagagtgaggTtagaaggagaagggTCaggagaatagcttagtagtagtTaggtaggagagtagcAgtagtt
aAagagaagtAcAagtAAaAagagtGaccagGTTCTTGAAGACGAAAGGGCCTCGTGATACGCCTATTTTTATAGGTTAA
TGTCATGATAATAATGGTTTCTTAGACGTCAGGTGGCACTTTTCGGGGAAATGTGCGCGGAACCCCTATTTGTTTATTTT
TCTAAATACATTCAAATATGTATCCGCTCATGAGACAATAACCCTGATAAATGCTTCAATAATATTGAAAAAGGAAGAGT
ATGAGTATTCAACATTTCCGTGTCGCCCTTATTCCCTTTTTTGCGGCATTTTGCCTTCCTGTTTTTGCTCACCCAGAAAC
GCTGGTGAAAGTAAAAGATGCTGAAGATCAGTTGGGTGCACGAGTGGGTTACATCGAACTGGATCTCAACAGCGGTAAGA
TCCTTGAGAGTTTTCGCCCCGAAGAACGTTTTCCAATGATGAGCACTTTTAAAGTTCTGCTATGTGGCGCGGTATTATCC
CGTGTTGACGCCGGGCAAGAGCAACTCGGTCGCCGCATACACTATTCTCAGAATGACTTGGTTGAGTACTCACCAGTCAC
AGAAAAGCATCTTACGGATGGCATGACAGTAAGAGAATTATGCAGTGCTGCCATAACCATGAGTGATAACACTGCGGCCA
ACTTACTTCTGACAACGATCGGAGGACCGAAGGAGCTAACCGCTTTTTTGCACAACATGGGGGATCATGTAACTCGCCTT
GATCGTTGGGAACCGGAGCTGAATGAAGCCATACCAAACGACGAGCGTGACACCACGATGCCTGCAGCAATGGCAACAAC
GTTGCGCAAACTATTAACTGGCGAACTACTTACTCTAGCTTCCCGGCAACAATTAATAGACTGGATGGAGGCGGATAAAG
TTGCAGGACCACTTCTGCGCTCGGCCCTTCCGGCTGGCTGGTTTATTGCTGATAAATCTGGAGCCGGTGAGCGTGGGTCT
CGCGGTATCATTGCAGCACTGGGGCCAGATGGTAAGCCCTCCCGTATCGTAGTTATCTACACGACGGGGAGTCAGGCAAC
TATGGATGAACGAAATAGACAGATCGCTGAGATAGGTGCCTCACTGATTAAGCATTGGTAACTGTCAGACCAAGTTTACT
CATATATACTTTAGATTGATTTAAAACTTCATTTTTAATTTAAAAGGATCTAGGTGAAGATCCTTTTTGATAATCTCATG
ACCAAAATCCCTTAACGTGAGTTTTCGTTCCACTGAGCGTCAGACCCCGTAGAAAAGATCAAAGGATCTTCTTGAGATCC
TTTTTTTCTGCGCGTAATCTGCTGCTTGCAAACAAAAAAACCACCGCTACCAGCGGTGGTTTGTTTGCCGGATCAAGAGC
TACCAACTCTTTTTCCGAAGGTAACTGGCTTCAGCAGAGCGCAGATACCAAATACTGTCCTTCTAGTGTAGCCGTAGTTA
GGCCACCACTTCAAGAACTCTGTAGCACCGCCTACATACCTCGCTCTGCTAATCCTGTTACCAGTGGCTGCTGCCAGTGG
CGATAAGTCGTGTCTTACCGGGTTGGACTCAAGACGATAGTTACCGGATAAGGCGCAGCGGTCGGGCTGAACGGGGGGTT
CGTGCACACAGCCCAGCTTGGAGCGAACGACCTACACCGAACTGAGATACCTACAGCGTGAGCTATGAGAAAGCGCCACG
CTTCCCGAAGGGAGAAAGGCGGACAGGTATCCGGTAAGCGGCAGGGTCGGAACAGGAGAGCGCACGAGGGAGCTTCCAGG
GGGAAACGCCTGGTATCTTTATAGTCCTGTCGGGTTTCGCCACCTCTGACTTGAGCGTCGATTTTTGTGATGCTCGTCAG
GGGGGCGGAGCCTATGGAAAAACGCCAGCAACGCGGCCTTTTTACGGTTCCTGGCCTTTTGCTGGCCTTTTGCTCACATG
TTCTTTCCTGCGTTATCCCCTGATTCTGTGGATAACCGTATTACCGCCTTTGAGTGAGCTGATACCGCTCGCCGCAGCCG
AACGACCGAGCGCAGCGAGTCAGTGAGCGAGGAAGCGGAAGAGCGCCTGATGCGGTATTTTCTCCTTACGCATCTGTGCG
GTATTTCACACCGCATATATGGTGCACTCTCAGTACAATCTGCTCTGATGCCGCATAGTTAAGCCAGTATACACTCCGCT
ATCGCTACGTGACTGGGTCATGGCTGCGCCCCGACACCCGCCAACACCCGCTGACGCGCCCTGACGGGCTTGTCTGCTCC
CGGCATCCGCTTACAGACAAGCTGTGACCGTCTCCGGGAGCTGCATGTGTCAGAGGTTTTCACCGTCATCACCGAAACGC
GCGAGGCAGCTGCGGTAAAGCTCATCAGCGTGGTCGTGAAGCGATTCACAGATGTCTGCCTGTTCATCCGCGTCCAGCTC
GTTGAGTTTCTCCAGAAGCGTTAATGTCTGGCTTCTGATAAAGCGGGCCATGTTAAGGGCGGTTTTTTCCTGTTTGGTCA
CTGATGCCTCCGTGTAAGGGGGATTTCTGTTCATGGGGGTAATGATACCGATGAAACGAGAGAGGATGCTCACGATACGG
GTTACTGATGATGAACATGCCCGGTTACTGGAACGTTGTGAGGGTAAACAACTGGCGGTATGGATGCGGCGGGACCAGAG
AAAAATCACTCAGGGTCAATGCCAGCGCTTCGTTAATACAGATGTAGGTGTTCCACAGGGTAGCCAGCAGCATCCTGCGA
TGCAGATCCGGAACATAATGGTGCAGGGCGCTGACTTCCGCGTTTCCAGACTTTACGAAACACGGAAACCGAAGACCATT
CATGTTGTTGCTCAGGTCGCAGACGTTTTGCAGCAGCAGTCGCTTCACGTTCGCTCGCGTATCGGTGATTCATTCTGCTA
ACCAGTAAGGCAACCCCGCCAGCCTAGCCGGGTCCTCAACGACAGGAGCACGATCATGCGCACCCGTGGCCAGGACCCAA
CGCTGCCCGAGATGCGCCGCGTGCGGCTGCTGGAGATGGCGGACGCGATGGATATGTTCTGCCAAGGGTTGGTTTGCGCA
TTCACAGTTCTCCGCAAGAATTGATTGGCTCCAATTCTTGGAGTGGTGAATCCGTTAGCGAGGTGCCGCCGGCTTCCATT
CAGGTCGAGGTGGCCCGGCTCCATGCACCGCGACGCAACGCGGGGAGGCAGACAAGGTATAGGGCGGCGCCTACAATCCA
TGCCAACCCGTTCCATGTGCTCGCCGAGGCGGCATAAATCGCCGTGACGATCAGCGGTCCAGTGATCGAAGTTAGGCTGG
TAAGAGCCGCGAGCGATCCTTGAAGCTGTCCCTGATGGTCGTCATCTACCTGCCTGGACAGCATGGCCTGCAACGCGGGC
ATCCCGATGCCGCCGGAAGCGAGAAGAATCATAATGGGGAAGGCCATCCAGCCTCGCGTCGCGAACGCCAGCAAGACGTA
GCCCAGCGCGTCGGCCGCCATGCCGGCGATAATGGCCTGCTTCTCGCCGAAACGTTTGGTGGCGGGACCAGTGACGAAGG
CTTGAGCGAGGGCGTGCAAGATTCCGAATACCGCAAGCGACAGGCCGATCATCGTCGCGCTCCAGCGAAAGCGGTCCTCG
CCGAAAATGACCCAGAGCGCTGCCGGCACCTGTCCTACGAGTTGCATGATAAAGAAGACAGTCATAAGTGCGGCGACGAT
AGTCATGCCCCGCGCCCACCGGAAGGAGCTGACTGGGTTGAAGGCTCTCAAGGGCATCGGTCGAGATCCCGGTGCCTAAT
GAGTGAGCTAACTTACATTAATTGCGTTGCGCTCACTGCCCGCTTTCCAGTCGGGAAACCTGTCGTGCCAGCTGCATTAA
TGAATCGGCCAACGCGCGGGGAGAGGCGGTTTGCGTATTGGGCGCCAGGGTGGTTTTTCTTTTCACCAGTGAGACGGGCA
ACAGCTGATTGCCCTTCACCGCCTGGCCCTGAGAGAGTTGCAGCAAGCGGTCCACGCTGGTTTGCCCCAGCAGGCGAAAA
TCCTGTTTGATGGTGGTTAACGGCGGGATATAACATGAGCTGTCTTCGGTATCGTCGTATCCCACTACCGAGATATCCGC
ACCAACGCGCAGCCCGGACTCGGTAATGGCGCGCATTGCGCCCAGCGCCATCTGATCGTTGGCAACCAGCATCGCAGTGG
GAACGATGCCCTCATTCAGCATTTGCATGGTTTGTTGAAAACCGGACATGGCACTCCAGTCGCCTTCCCGTTCCGCTATC
GGCTGAATTTGATTGCGAGTGAGATATTTATGCCAGCCAGCCAGACGCAGACGCGCCGAGACAGAACTTAATGGGCCCGC
TAACAGCGCGATTTGCTGGTGACCCAATGCGACCAGATGCTCCACGCCCAGTCGCGTACCGTCTTCATGGGAGAAAATAA
TACTGTTGATGGGTGTCTGGTCAGAGACATCAAGAAATAACGCCGGAACATTAGTGCAGGCAGCTTCCACAGCAATGGCA
TCCTGGTCATCCAGCGGATAGTTAATGATCAGCCCACTGACGCGTTGCGCGAGAAGATTGTGCACCGCCGCTTTACAGGC
TTCGACGCCGCTTCGTTCTACCATCGACACCACCACGCTGGCACCCAGTTGATCGGCGCGAGATTTAATCGCCGCGACAA
TTTGCGACGGCGCGTGCAGGGCCAGACTGGAGGTGGCAACGCCAATCAGCAACGACTGTTTGCCCGCCAGTTGTTGTGCC
ACGCGGTTGGGAATGTAATTCAGCTCCGCCATCGCCGCTTCCACTTTTTCCCGCGTTTTCGCAGAAACGTGGCTGGCCTG
GTTCACCACGCGGGAAACGGTCTGATAAGAGACACCGGCATACTCTGCGACATCGTATAACGTTACTGGTTTCACATTCA
```

```
CCACCCTGAATTGACTCTCTTCCGGGCGCTATCATGCCATACCGCGAAAGGTTTTGCGCCATTCGATGGTGTCCGGGATC
TCGACGCTCTCCCTTATGCGACTCCTGCATTAGGAAGCAGCCCAGTAGTAGGTTGAGGCCGTTGAGCACCGCCGCCGCAA
GGAATGGTGCATGCAAGGAGATGGCGCCCAACAGTCCCCCGGCCACGGGGCCTGCCACCATACCCACGCCGAAACAAGCG
CTCATGAGCCCGAAGTGGCGAGCCCGATCTTCCCCATCGGTGATGTCGGCGATATAGGCGCCAGCAACCGCACCTGTGGC
GCCGGTGATGCCGGCCACGATGCGTCCGGCGTAGAGGATCGAGATCTCGATCCCGCGAAATTAATACGACTCACTATAGG
GGAATTGTGAGCGGATAACAATTCCCCTCTAGAAATAATTTTGTTTAACTTTAAGAAGGAGATATAcatatgAAAAAGGG
GCACCACCATCATCACCACGGGTCTGAACGCACAGGTACTCAACCCCTTGGAGTCCAGGGCTTGACAGAGGAACAGCGTA
TGATGATCCGTGAGTTAATGGATGCGCAGATGAAAACTTTCGATACGACTTTTTCTCACTTCAAGAACTTTCGTCTGCCG
GGGGTTCTGTCTTCCGGCTGCGAGTTACCCGAATCGTTACAGGCGCCCTCACGCGAAGAAGCCGCAAAATGGAGTCAAGT
TCGCAAAGATTTATGCTCTTTAAAAGTGTCCTTGCAATTACGTGGCGAAGACGGTTCCGTTTGGAACTATAAGCCGCCAG
CGGACAGCGGCGGAAAAGAAATCTTCAGTCTGTTGCCGCACATGGCCGATATGTCCACTTACATGTTTAAGGGAATTATC
TCGTTTGCTAAGGTTATTTCATACTTCCGCGATTTACCTATTGAGGACCAAATTTCCCTGTTAAAAGGGGCCGCCTTTGA
ATTGTGTCAATTGCGCTTCAATACCGTGTTTAATGCTGAAACTGGCACTTGGGAGTGTGGACGCTTAAGCTATTGCTTGG
AGGATACCGCAGGAGGATTTCAACAACTTCTTTTGGAGCCAATGCTGAAATTTCATTATATGTTGAAAAAGTTGCAGTTG
CATGAAGAAGAGTATGTGTTGATGCAAGCGATCTCTCTTTTCTCGCCCGATCGTCCAGGCGTGCTGCAGCACCGTGTTGT
GGACCAGCTTCAGGAGCAGTTCGCTATCACGTTAAAGAGTTATATCGAGTGTAATCGTCCGCAACCAGCACATCGTTTTC
TGTTCTTAAAAATTATGGCTATGTTGACTGAGTTGCGTAGTATTAATGCACAGCACACCCAGCGCTTACTGCGCATTCAG
GATATTCATCCATTCGCAACACCCCTGATGCAAGAATTATTCGGAATTACAGGCTCGCTGGTGCCCCGCGGCTCGTCATC
TCACTCGTCGCTGACTGAACGTCACAAGATTCTGCATCGTTTGTTGCAAGAAGGGTCGCCTTCATGAaagcttATCGATG
ATAAGCTGTCAAACATGAGAA
```

```
Features :
Amp prom        : [251 : 279 - CW]
AmpR            : [519 : 1178 - CW]
ColE1 origin    : [1330 : 1958 - CW]
lacI            : [4964 : 3873 - CCW]
T7              : [5342 : 5361 - CW]
LacO            : [5361 : 5383 - CW]
Met start       : [5430 : 5432 - CW]
His Tag         : [5442 : 5459 - CW]
PXR-SRC1        : [5463 : 6464 - CW]
Thrombine site  : [6378 : 6395 - CW]
Stop            : [6465 : 6467 - CW]
```

S1.2. Auto-induction growth media and culture protocol

A. Medium preparation

Just before culture, mix 500 ml of ZYM 50-52, 10 ml of 50X M, 1 ml of $MgSO_4$ 1 M, 10 ml of 50X 5052, 100 µl of metal mix, in a 2 l flask.

ZYM 50-52 (500 ml), resuspend powders in water, sterilize, and store at room temperature.

Nutrients	Final concentration	Mass (g)
NZ amine	1%	5.0
Yeast Extract	0.5%	2.5

50X M (500 ml), resuspend powders in water, sterilize, and store at room temperature.

Salts	MW (g mol^{-1})	Final concentration (M)	Mass (g)
Na_2HPO_4	141.96	1.25	88.7
KH_2PO_4	136.08	1.25	85.1
NH_4Cl	53.49	2.5	66.9
Na_2SO_4	142.04	0.25	17.7

50X 5052 (500 ml), resuspend powders in water, sterilize, and store at room temperature.

Sugars	Final concentration	Mass (g)
Glycerol	25%	125.0
Glucose	2.5%	12.5
Lactose	10%	50.0

Metal mix (5 ml), resuspend powders in water, filter on 0.22 μm in a sterile tube of 15 ml, and store at room temperature.

Metal	MW (g mol^{-1})	Final concentration (mM)	Mass (mg)
$FeCl_3$	162.20	50	121.6
$CaCl_2$	110.98	20	48.7
$MnCl_2$	125.84	10	18.9
$ZnSO_4$	161.47	10	24.2
$CoCl_2$	129.84	2	3.9
$CuCl_2$	134.45	2	4.0
$NiCl_2$	129.59	2	3.9

H_3BO_3	61.83	2	1.8
Na_2SeO_3	172.94	2	5.2
Na_2MoO_2	173.93	2	5.2

B. Culture protocol

Subculture

Inoculate 50 ml of LB medium supplemented with the proper antibiotic with freshly transformed colonies. Incubate at 37°C, 220 rpm overnight.

Culture

Prepare the medium as previously described and add the proper antibiotic. Inoculate with the subculture to reach an initial OD_{600} of 0.15. Incubate at 37°C, 220 rpm for 4h and then change the temperature at 25°C for the night.

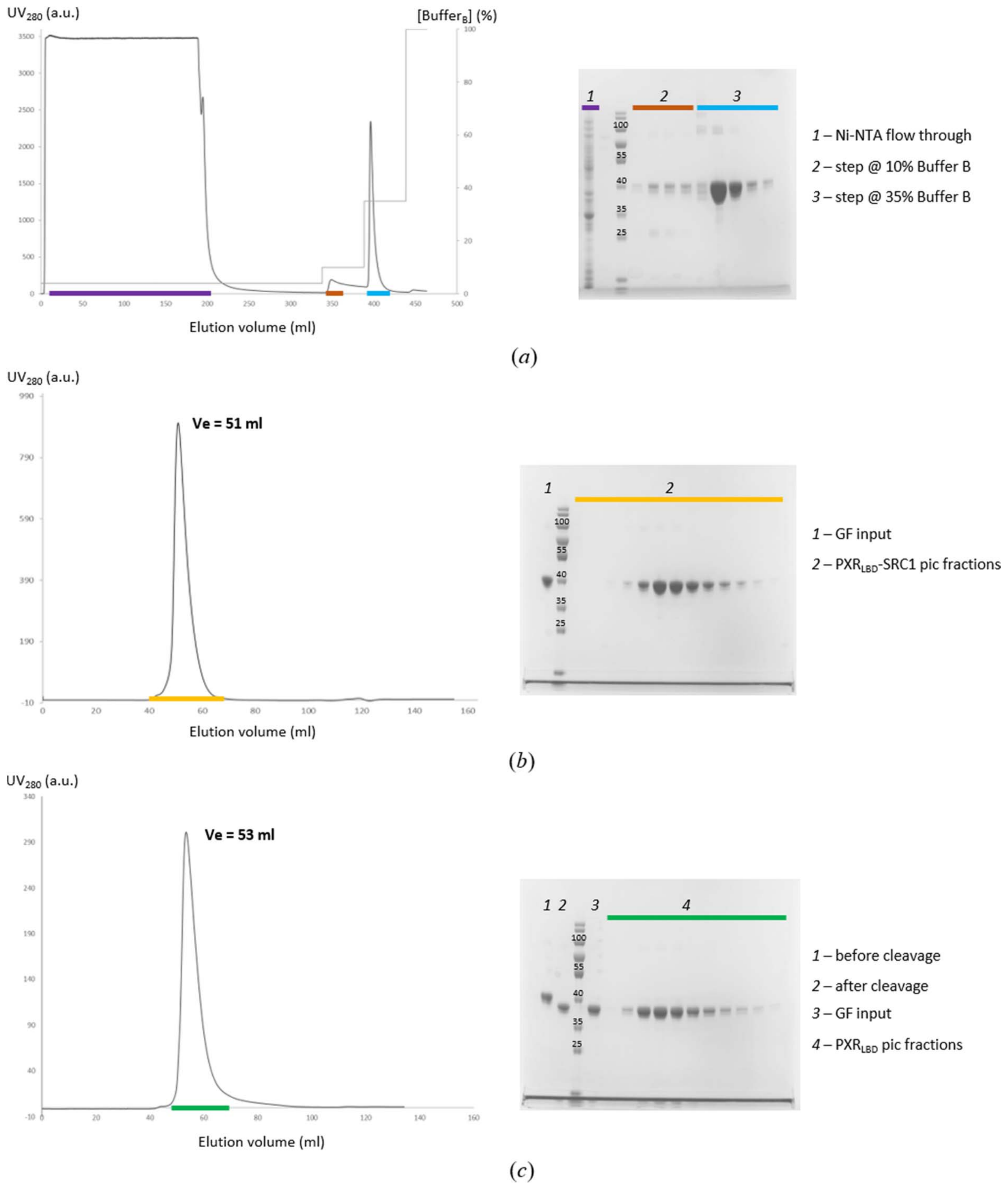

Figure S1 A modular purification protocol to obtain the PXR_{LBD} with or without the SRC-1 moiety. Chromatograms and SDS-PAGE for the affinity (*a*) and the gel filtration (*b*,*c*) steps. In each case, the corresponding fractions are indicated by a color bar. The thrombin cleavage control is visible on the gel in (*c*).
